# Healthy lifestyle knowledge and age at hypertension diagnosis: a primary health care based survey in Bangladesh

**DOI:** 10.1038/s41371-025-01019-3

**Published:** 2025-04-25

**Authors:** Md Monirul Islam, Md. Safayet Hossain, Md. Mizanur Rahman, Ryota Nakamura, Motohiro Sato

**Affiliations:** 1 Global Public Health Research Foundation, Dhaka, Bangladesh; 2 Centre for Policy Studies, Cork University Business School, University College Cork, Cork, Ireland; 3 Department of Statistics, Comilla University, Comilla, Bangladesh; 4 Hitotsubashi Institute for Advanced Study, Hitotsubashi University, Tokyo, Japan; 5 Graduate School of Economics, Hitotsubashi University, Tokyo, Japan; 6 Department of Global Health & Development, London School of Hygiene & Tropical Medicine, London, UK

**Keywords:** Lifestyle modification, Hypertension

## Abstract

This study examined the relationship between knowledge of healthy lifestyles and the age of hypertension diagnosis among hypertensive individuals within Bangladeshi rural population. This cross-section study was conducted among hypertensive adults (18–80 years) in a rural population. We obtained data from 3600 adults with hypertension from 40 randomly selected community pharmacies. We gathered data on demographics, health knowledge, and measured vital signs, including hypertension diagnosis year. Multinomial logistic regression analysis was used to identify the lifestyle and knowledge factors about hypertension with the age of diagnosis of hypertension. The mean age of hypertension diagnosis was 45.84 years. The mean age of hypertension diagnosis of male participants was higher than female (48.1 vs 44.4 years). Our study found that males and individuals with primary education are more likely to receive a later hypertension diagnosis (odds ratio = 2.32; 95% confidence interval: 1.75–3.10 and odds ratio = 5.96; 95% confidence interval: 3.09–11.48 respectively) for those aged ≥65. The poorest and those lacking physical exercise faced higher odds of later diagnosis (odds ratio = 2.20; 95% confidence interval: 1.53–3.15 and odds ratio = 2.37; 95% confidence interval: 1.78–3.17 respectively). Conversely, a family history of hypertension reduces the odds (odds ratio = 0.38; 95% confidence interval: 0.27–0.55). Increased knowledge of healthy lifestyle factors and engagement with health-related media correlate with later diagnosis, highlighting the influence of education and awareness on hypertension detection age. Our study reveals that knowledge of a healthy lifestyle is associated with the age of hypertension diagnosis. Targeting specific age groups based on health education programs may reduce hypertension-related complications.

## Introduction

Hypertension is a leading risk factor for disease burden within low and middle-income countries (LMICs) populations due to higher prevalence than the high-income countries [1]. Uncontrolled hypertension leads to cardiovascular disease, kidney disease, and stroke [2]. Previous studies referred to hypertension as a “silent killer” because of its asymptomatic and persistent nature [3]. Although the etiology of uncontrolled hypertension is considered as multifactorial and involves both biological and behavioral determinants, lifestyle plays an important role in the development and management of hypertension [4].

The evidence from previous studies suggests that diet, physical activity, and adherence to treatment are essential for hypertension control, which ultimately prevents its long-term complications as well [5]. Previous studies mentioned that persons who practice healthy lifestyles (regular physical activity, taking a balanced diet, and avoiding tobacco) are more likely to have better-controlled blood pressure levels [6]. Furthermore, clinical trials have consistently reported that a reduction in the dietary intake of sodium favorably affects blood pressure (BP) among older adults [7]. Although, practicing these healthy lifestyle behaviors depends on a persons awareness and understanding of hypertension and its associated risks. Also, hypertensive individuals who are aware of the condition are better equipped to make informed decisions that improve their health outcomes [8]. Early detection, treatment and good control of hypertension are associated with significant positive effects on health and economic aspects [9].

As like many other LMICs, in Bangladesh, there is a lack of adequate knowledge and awareness about hypertension within rural populations because of difficult access to healthcare services and limited educational programs [10]. Therefore, a significant portion of the population does not recognize the importance of monitoring their blood pressure, adhering to medications, or adopting a healthy lifestyle [11]. The lack of knowledge and awareness contributes to late diagnosis of hypertension which hinders effective disease management. Moreover, the patients do not fully understand the condition or the importance of lifestyle modifications, as a result, they are less likely to adhere to treatment plans, leading to higher rates of uncontrolled blood pressure. Moreover, non-adherence to antihypertensive medications is related to poor health knowledge.

Previous studies mentioned that individuals with inadequate knowledge about their condition are more likely to miss doses, stop receiving medications or abstain from visiting the doctor [12, 13]. These conditions may increase blood pressure as well as progress the risk of complications such as stroke, heart disease, and kidney failure [2]. On the other hand, those who are aware of their health condition tend to be more proactive in managing their health.

Although it has been well documented that there is a relationship between lifestyle and hypertension management, little is known about how healthy lifestyle knowledge influences the timing of hypertension diagnosis. As to prevent the disease progression and lower the risk of complication it is important to diagnose the hypertension as early as possible. In the context of rural Bangladesh, where healthcare services are often limited, understanding how lifestyle knowledge impacts the age of diagnosis could provide valuable insights for public health interventions. Since there is a higher prevalence of hypertension and limitations in the knowledge among the rural population in Bangladesh [14], it’s important to evaluate how health knowledge influences hypertension diagnosis. Therefore, this study aimed to identify the relationship between knowledge of healthy lifestyles and age of hypertension diagnosis among them. By examining the association between health knowledge and the age of diagnosis, this research could inform future strategies to promote early detection and improve hypertension management in rural communities.

## Methods

### Study design and participants

This cross-sectional study was part of an ongoing community pharmacy-based randomized control study for hypertension control in Bangladesh (Trial registration number: ClinicalTrials.gov NCT06148142). Due to logistical support, we selected Rangpur and Chuadanga districts of Bangladesh. A randomization was then conducted to select one subdistricts (Pirganj and Alamdanga) from each district. In line with our study aims, we only included participants from the rural areas of Pirganj and Alamdanga Upazila. The two-stage random selection process was meticulously devised to ensure that the chosen districts reflect a diverse socio-demographic landscape. This approach facilitates random selection while maintaining a broad representation within the districts. A total of 3600 adults with hypertension were chosen from 40 randomly selected community pharmacies in two Upazilas, based on the following inclusion criteria: (1) aged ≥18 years, (2) diagnosed with hypertension (systolic blood pressure ≥140 mmHg and/or diastolic blood pressure ≥90 mmHg, and/or taking antihypertensive drugs), (3) currently residing in the area, (4) having a mobile phone, (5) speaking the local language, and (6) able to provide consent to participate. We excluded participants who: (1) are pregnant, (2) have advanced diseases (e.g., cancer, severe kidney/renal disease, heart failure, severe pulmonary dysfunction, severe neurological dysfunction, etc.).

### Data collection and measurements

All participants underwent mandatory questionnaire-based, physical, and clinical measurements. Trained research personnel conducted the surveys in the participants’ native language and recorded their blood pressure and other physical measurements. The questionnaire gathered information on socio-demographics, such as age, sex, religion, marital status, family type, income, educational attainment, and type of work performed, as well as lifestyle-related behaviours, including tobacco use, alcohol consumption, consumption of healthy food, and physical activity. BP was measured using a digital automatic BP monitor in a seated position at the right hand, where possible, with the arm supported at the heart level. BP was measured twice: once at the beginning of the survey and again at the end, with a minimum interval of 15 min between measurements. The average of the two readings was used to determine hypertensive status. A detailed description of the data collection is reported in our study protocol [15].

### Outcome variables: age at diagnosis of hypertension

Outcome variables for this study was age at diagnosis of hypertension. All the survey participants were asked the age at which they were first informed by a doctor or other health professional that they had hypertension or high blood pressure. In cases where participants provided the total number of years since their diagnosis, the duration of hypertension was then computed by subtracting the age at the first diagnosis from their current age. The diagnosis of hypertension was categorized by age groups: <45 years, 45–54 years, 55–64 years, and ≥65 years to align with existing literature [16, 17].

### Predictor variables: knowledge about healthy lifestyle

Participants were asked a series of questions about hypertension-related lifestyle knowledge with responses recorded as either ‘yes’ or ’no’. The questions were: (1) do you know extra salt intake is harmful to health? ; (2) do you know obesity can cause hypertension? ; (3) do you know a sedentary lifestyle is one of the reasons for hypertension?; (4) do you know alcohol consumption can cause hypertension?; (5) do you know daily 30 min of walking/running daily can reduce blood pressure?; (6) do you know sleep deprivation/ less sleep/ going to bed late at night can cause hypertension or increase blood pressure?; (7) do you know adherence to daily drug doses reduces hypertension/ helps maintain blood pressure?; (8) do you know monitoring blood pressure helps maintain blood pressure?; (9) do you watch health-related TV programs?; (10) do you read health-related newspapers? (Yes/No).

### Ethical approval

The study was approved by the ethics committee of Hitotsubashi University (Approval Number: 2023D016). Prior to data collection, informed consent was obtained from the study participants and community pharmacists after explaining the voluntary nature of participation. They were also informed of their right to withdraw from the study at any stage. We also confirmed that their data will be kept confidential, and only anonymous data will be used for analysis and publication.

### Statistical analyses

To acquire the basic information on the socio-demographic characteristics of the respondents, a descriptive analysis was performed. For categorical variables, the frequency with percentages was recorded and for continuous variables, the mean with standard deviation was reported. For visualisation and a better understanding of the results, bar charts and line graphs were used. ANOVA tests were performed to obtain the association between the demographic characteristics of the population and the mean age of diagnosis of hypertension. To assess the mean age at which hypertension is diagnosed, the mean and standard deviation were calculated. Additionally, Pearson chi-square tests were used to measure the association between the categorical variables (covariates) and age of diagnosis of hypertension. The decision has been taken based on the *p*-value. In all analyses, *p*-value < 0.05 was considered statistically significant. Multinomial logistic regression was used to obtain unadjusted odds ratios to identify the lifestyle and knowledge factors with the age of diagnosis of hypertension. Odds ratio (OR) with 95% confidence interval (CI) was reported for the individual variable. Finally, the adjusted multinomial model was performed to determine the factors that combinedly associated with the age of hypertension diagnosis. All statistical analyses were performed by Stata v17.0.

## Results and findings

### Characteristics of participants

**Fig. 1:**
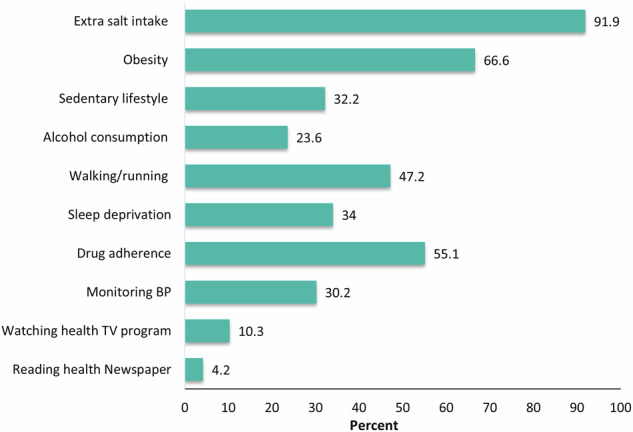
Knowledge about healthy lifestyles among all participants, Bangladesh.

Our study comprised 3600 hypertensive adults. Table 1 shows the demographic characteristics of the study population. Our analysis shows that the majority (27.8%) of participants were between the ages of 50 and 59 years and mostly were male (61.4%). Among them, 50.2% had no formal education and nearly a third (28.9%) had a family history of hypertension. Fig. 1 shows the percentage of participants with knowledge about various lifestyle factors related to hypertension. Where 91.9% of participants knew the harmful effects of extra salt intake and 66.6% of respondents knew that obesity can cause hypertension. However, only 10.3% watch health-related TV programs, and only 4.2% read health-related newspapers. Moreover, all participants education levels and knowledge about healthy lifestyles are shown in Table 2.

**Table 1:** Study characteristics of the population, Bangladesh.

Demographic Characteristics	N	%
Age, years		
≤30	103	2.9
31–39	320	8.9
40–49	801	22.3
50–59	999	27.8
60–69	938	26.1
≥70	439	12.2
Sex		
Male	2209	61.4
Female	1391	38.6
Education		
No education	1806	50.2
Primary	873	24.3
Secondary	545	15.1
Higher	376	10.4
Body Mass Index (kg/m2)		
Underweight	248	6.9
Normal Weight	1776	49.3
Overweight	1228	34.1
Obese	348	9.7
Blood pressure		
Poor	1025	28.5
Intermediate	2186	60.7
Ideal	389	10.8
Expenditure quintile		
Q1 (poorest)	962	26.7
Q2	1006	27.9
Q3	729	20.2
Q4	592	16.4
Q5 (richest)	311	8.6
Physical Exercise		
Yes	1940	46.1
No	1660	53.9
Smoking status		
Non-smoker	3182	88.4
Ex-smoker	69	1.9
Current-smoker	349	9.7
Family history of Hypertension		
Yes	1040	28.9

Values are n (%).

**Table 2:** Education Level and knowledge about healthy lifestyles among all participants, Bangladesh.

Knowledge	No education	Primary	Secondary	Higher
Do you know extra salt intake is harmful for health?	1623 (89.9)	804 (96.3)	519 (95.2)	362 (96.3)
Do you know obesity can cause hypertension?	1039 (57.5)	582 (89.6)	441 (80.9)	337 (89.6)
Do you know sedentary lifestyle is one of the reasons of hypertension	464 (25.7)	271 (51.6)	231 (42.4)	194 (51.6)
Do you know alcohol consumption can cause hypertension?	328 (18.2)	182 (42.8)	179 (32.8)	161 (42.8)
Do you know daily 30 min walking/running can reduce hypertension?	706 (39.1)	437 (65.4)	311 (57.1)	246 (65.4)
Do you know sleep deprivation/ less sleep/ going bed late at night can increase blood pressure?	564 (31.2)	283 (43.9)	212 (38.9)	165 (43.9)
Do you know adherence to daily drug dose reduces hypertension/ helps maintaining	892 (49.4)	487 (72.3)	334 (61.3)	272 (72.3)
Do you know monitoring blood pressure helps maintaining blood pressure?	497 (27.5)	225 (44.7)	198 (36.3)	168 (44.7)
Do you watch health related TV program?	62 (3.4)	62 (37)	107 (19.6)	139 (37)
Do you read health related newspapers?	3 (0.2)	6 (29)	34 (6.2)	109 (29)

Values are n (%).

### Age of hypertension diagnosis and lifestyle factors

Table 3 indicates the demographic characteristics of the population based on the age of diagnosis of hypertension. The age of diagnosis of hypertension varies significantly across demographic and lifestyle factors. Sex, education, BMI, Blood pressure, physical activity, and family history of hypertension are significantly associated with the mean age of diagnosis of hypertension. Moreover, knowledge of hypertension including extra salt intake, obesity, sedentary lifestyle, walking/running, watching health-related TV programs, and reading health newspapers have significant associations with the mean age of diagnosis. Males tend to be diagnosed later (on average 48.1 years) than females (44.4 years). Also, people with no education are diagnosed at an older age (49.0 years) compared to those with education (41.3 years).

**Table 3:** Demographic characteristics of the population based on the age of diagnosis of hypertension, Bangladesh.

Demographic Characteristics	Age of diagnosis (years)	*P*-value
	Mean ± SD	
All participants (*N* = 3600)		
Total	45.8 ± 11.5	
Sex		0.000
Female	44.4 ± 11.0	
Male	48.1 ± 11.7	
Education		0.021
No education	49.0 ± 10.7	
Primary	43.8 ± 11.1	
Secondary	41.9 ± 11.7	
Higher	41.3 ± 11.1	
Body Mass Index (kg/m2)		0.048
Underweight	52.0 ± 11.4	
Normal Weight	47.3 ± 11.5	
Overweight	43.6 ± 10.7	
Obese	41.9 ± 11.1	
Blood pressure		0.004
Poor	44.8 ± 11.0	
Intermediate	46.5 ± 11.7	
Ideal	44.8 ± 11.0	
Expenditure quintile		0.423
Q1 (poorest)	48.1 ± 11.3	
Q2	45.0 ± 11.5	
Q3	45.5 ± 11.1	
Q4	44.7 ± 11.5	
Q5 (richest)	44.5 ± 11.6	
Physical Exercise		0.000
Yes	44.4 ± 11.0	
No	47.6 ± 11.8	
Smoking status		0.323
Non-smoker	45.7 ± 11.4	
Ex-smoker	53.7 ± 10.2	
Current-smoker	45.4 ± 11.4	
Family history of Hypertension		0.000
Yes	43.0 ± 11.2	
No	47.0 ± 11.4	
Knowledge about hypertension		
Extra salt intake		0.000
Yes	45.6 ± 11.4	
No	48.0 ± 12.0	
Obesity		0.000
Yes	45.1 ± 11.4	
No	47.3 ± 11.5	
Sedentary lifestyle		0.004
Yes	45.0 ± 11.5	
No	46.2 ± 11.4	
Alcohol consumption		0.523
Yes	46.1 ± 11.7	
No	45.8 ± 11.4	
Walking/running		0.023
Yes	45.4 ± 11.4	
No	46.3 ± 11.5	
Sleep deprivation		0.839
Yes	45.8 ± 11.7	
No	45.9 ± 11.4	
Drug adherence		0.627
Yes	45.9 ± 11.5	
No	45.7 ± 11.5	
Monitoring BP		0.271
Yes	45.5 ± 11.7	
No	46.0 ± 11.4	
Watching health TV program		0.000
Yes	42.5 ± 11.6	
No	46.2 ± 11.4	
Reading health Newspaper		0.000
Yes	42.4 ± 10.1	
No	46.0 ± 11.5	

*HT* hypertension, *TV* television.

**Fig. 2:**
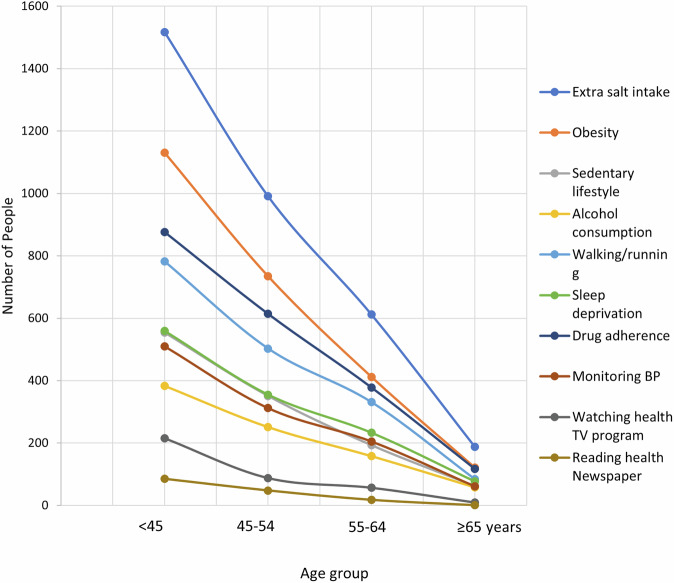
Knowledge about healthy lifestyles across age groups, Bangladesh.

Figure 2 denotes that, participants diagnosed at higher age have less knowledge about healthy lifestyles. Age group-based diagnosis of hypertension and association with lifestyle-related knowledge is presented in Table 4. Females are more likely to be diagnosed before 45 years (51.1%) compared to males (36.2%). Whereas males are more prone to be diagnosed with later age, around 32% of them are diagnosed between 45–54 years, and 23.8% are diagnosed between 45–54 years. Moreover, higher education is associated with earlier detection. For example, 61.2% of those with higher education were diagnosed before 45 years of age. Obesity is also associated with earlier diagnosis, with 62.6% under 45 years, old compared to normal weight. Individuals who engage in physical exercise are diagnosed earlier (50.3% under 45 years). Individuals with a family history of hypertension show an earlier diagnosis trend, with 55.9% diagnosed before 45 years. Additionally, those knowledgeable about extra salt intake and obesity are similarly diagnosed early (before 45 years). Health media engagement also influences early diagnosis; 58.1% of those who watch health TV programs and 55.9% of those who read health newspapers are diagnosed before 45 years, with around 24–32% diagnosed between 45 and 54 years.

**Table 4:** Age group-based diagnosis of hypertension and association with lifestyle-related knowledge, Bangladesh.

	Age of diagnosis of hypertension (frequency, %)				
Characteristics	<45 years (*n* = 1632)	45–54 years (*n* = 1068)	55–64 years (*n* = 682)	≥65 years (*n* = 218)	*P*-value
Gender					0.000
Female	1128 (51.1)	623 (28.2)	351 (15.9)	107 (4.8)	
Male	504 (36.2)	445 (32.0)	331 (23.8)	111 (8.0)	
Education					0.000
No education	637 (35.3)	579 (32.1)	425 (23.5)	165 (9.1)	
Primary	445 (51.0)	263 (30.1)	137 (15.7)	28 (3.2)	
Secondary	320 (58.7)	131 (24.0)	79 (14.5)	15 (2.8)	
Higher	230 (61.2)	95 (25.3)	41 (10.9)	10 (2.7)	
Body Mass Index (kg/m^2^)					0.000
Underweight	66 (26.6)	64 (25.8)	80 (32.3)	38 (15.3)	
Normal Weight	689 (38.8)	579 (21.6)	383 (21.6)	125 (7.0	
Overweight	659 (53.7)	348 (28.3)	181 (14.7)	40 (3.3)	
Obese	218 (62.6)	77 (22.1)	38 (10.9)	15 (4.3)	
Blood pressure (mmHg)					0.000
Poor	521 (50.8)	296 (28.9)	157 (15.3)	51 (5.0)	
Intermediate	922 (42.2)	650 (29.7)	470 (21.5)	144 (6.6)	
Ideal	189 (48.6)	122 (31.2)	55 (14.1)	23 (5.9)	
Expenditure quintile					0.000
Q1 (poorest)	344 (35.8)	314 (32.6)	227 (23.6)	77 (8.0)	
Q2	479 (47.1)	300 (29.8)	177 (17.6)	50 (5.0)	
Q3	342 (46.9)	216 (29.6)	130 (17.8)	41 (5.6)	
Q4	304 (51.4)	159 (26.9)	99 (16.7)	30 (5.1)	
Q5 (richest)	163 (52.4)	79 (25.4)	49 (15.8)	20 (6.4)	
Physical Exercise					0.000
Yes	976 (50.3)	568 (29.3)	312 (16.1)	84 (4.3)	
No	656 (39.5)	500 (30.1)	370 (22.3)	134 (8.1)	
Smoking status					0.000
Non-smoker	1461 (46.0)	938 (29.5)	591 (18.6)	192 (6.0)	
Ex-smoker	14 (20.3)	17 (24.6)	27 (39.1)	11 (15.9)	
Current-smoker	157 (45.0)	113 (32.4)	64 (18.3)	15 (4.3)	
Family history of HT					0.000
Yes	581 (55.9)	281 (27.0)	140 (13.5)	38 (3.7)	
Knowledge about HT					
Extra salt intake	1517 (45.9)	991 (30.0)	612 (18.5)	188 (5.7)	0.001
Obesity	1131 (47.1)	735 (30.6)	412 (17.2)	121 (5.0)	0.000
Sedentary lifestyle	554 (47.8)	351 (30.3)	193 (16.6)	62 (5.34)	0.034
Alcohol consumption	383 (45.1)	251 (29.5)	158 (18.6)	58 (6.8)	0.757
Walking/running	782 (46.0)	503 (29.6)	331 (19.5)	84 (5.0)	0.062
Sleep deprivation	559 (45.7)	355 (29.0)	233 (19.0)	77 (6.3)	0.919
Drug adherence	876 (44.1)	614 (30.9)	378 (19.0)	117 (5.9)	0.260
Monitoring BP	510 (46.9)	312 (28.7)	205 (18.8)	61 (5.6)	0.602
Watching health TV program	215 (58.1)	88 (23.8)	57 (15.4)	10 (2.7)	0.000
Reading health Newspaper	85 (55.9)	48 (31.6)	18 (11.8)	1 (0.7)	0.001

Values are n (%).

*HT* hypertension, *TV* television.

### Determinants of hypertension diagnosis age

As per Table 5, the findings indicate that several factors are associated with the age of hypertension diagnosis and knowledge of a healthy lifestyle. Males have a higher likelihood of being diagnosed later in life, with an odds ratio (OR) of 2.32 (95% CI: 1.75–3.10) for those aged 65 and above compared to females. Individuals with primary education compared to no education have significantly higher odds of later diagnosis (OR = 5.96, 95% CI: 3.09–11.48) for those aged ≥65 years than those aged <45 years. In terms of socio-economic factors, the poorest individuals (Q1) have higher odds of later diagnosis, especially for the 55–64 group (OR = 2.20, 95% CI: 1.53–3.15). Lack of physical exercise is associated with a later diagnosis (OR = 2.37, 95% CI: 1.78–3.17) for those aged ≥65. Family history of hypertension is protective, with odds decreasing with age, (OR: 0.38, 95% CI: 0.27–0.55) for those ≥65. Additionally, knowledge about healthy lifestyle factors improves with age at diagnosis. Particularly, those who recognize the dangers of extra salt intake have increased odds of a later diagnosis, especially in the ≥65 year’s group. Similarly, awareness of the health risks of obesity (OR = 1.81, 95% CI: 1.36–2.41 for ≥65 years) and the importance of physical activity like walking and running (OR = 1.47, 95% CI: 1.10–1.96 for ≥65 years) shows a positive association with later diagnosis in older individuals. Furthermore, engagement with health-related media, such as watching health programs on TV (OR = 3.15, 95% CI: 1.65–6.04) and reading health newspapers (OR = 10.37, 95% CI: 1.63–65.92) is associated with significantly higher odds of a delayed diagnosis of hypertension.

**Table 5:** Association between age of diagnosis of hypertension and knowledge about healthy lifestyle, Bangladesh.

	Age of diagnosis of hypertension (Unadjusted OR, 95% CI)		
Characteristics	45–54 years	55–64 years	≥65 years
Gender			
Female	1.00		
Male	1.60 (1.36–1.88)	2.11 (1.75–2.54)	2.32 (1.75–3.10)
Education			
No education	2.20 (3.74–5.96)	3.74 (2.63–5.33)	5.96 (3.09–11.48)
Primary	1.43 (1.73–1.45)	1.73 (1.18–2.53)	1.45 (0.69–3.03)
Secondary	0.99 (1.39–1.08)	1.39 (0.92–2.09)	1.08 (0.48–2.44)
Higher	1.00		
Body Mass Index (kg/m^2^)			
Underweight	1.15 (0.80–1.66)	2.18 (1.53–3.09)	3.17 (2.04–4.94)
Overweight	0.62 (0.53–0.75)	0.49 (0.40–0.61)	0.33 (0.23–0.49)
Obese	0.42 (0.32–0.56)	0.55 (0.22–0.45)	0.18 (0.22–0.66)
Normal Weight	1.00		
Blood pressure (mmHg)			
Poor	0.88 (0.67–1.15)	1.04 (0.73–1.47)	0.80 (0.48–1.35)
Intermediate	1.09 (0.85–1.10)	1.75 (1.27–2.41)	1.28 (0.80–2.05)
Ideal	1.00		
Expenditure quintile			
Q1 (poorest)	1.88 (1.38–2.56)	2.20 (1.53–3.15)	1.82 (1.08–3.09)
Q2	1.29 (0.95–1.75)	1.23 (0.86–1.77)	0.85 (0.49–1.47)
Q3	1.30 (0.95–1.79)	1.26 (0.87–1.85)	0.98 (0.55–1.72)
Q4	1.08 (0.78–1.50)	1.08 (0.73–1.60)	0.80 (0.44–1.46)
Q5 (richest)	1.00		
Physical Exercise			
No	1.31 (1.76–2.37)	1.76 (1.47–2.11)	2.37 (1.78–3.17)
Yes	1.00		
Smoking status			
Non-smoker	1.00		
Ex-smoker	1.89 (0.92–3.85)	4.77 (2.48–9.16)	5.98 (2.68–13.36)
Current-smoker	1.12 (0.86–1.45)	1.01 (0.74–1.37)	0.73 (0.42–1.26)
Family history of hypertension			
Yes	0.66 (0.55–0.77)	0.47 (0.38–0.58)	0.38 (0.27–0.55)
No	1.00		
Knowledge			
Extra salt intake^a^	1.02 (0.76–1.38)	1.51 (1.10–2.06)	2.10 (1.37–3.23)
Obesity^a^	1.02 (0.87–1.20)	1.48 (1.23–1.78)	1.81 (1.36–2.41)
Sedentary lifestyle^a^	1.04 (0.89–1.23)	1.30 (1.07–1.58)	1.29 (0.95–1.77)
Alcohol consumption^a^	1.00 (0.83–1.20)	1.02 (0.82–1.26)	0.85 (0.61–1.17)
Walking/running^a^	1.03 (0.89–1.21)	0.98 (0.82–1.17)	1.47 (1.10–1.96)
Sleep deprivation^a^	1.05 (0.89–1.23)	1.00 (0.83–1.21)	0.95 (0.71–1.28)
Drug adherence^a^	0.86 (0.73–1.00)	0.93 (0.78–1.12)	1.00 (0.75–1.33)
Monitoring BP^a^	1.10 (0.93–1.30)	1.06 (0.87–1.28)	1.17 (0.85–1.60)
Watching health TV program^a^	1.69 (1.30–2.19)	1.66 (1.22–2.60)	3.15 (1.65–6.04)
Reading health Newspaper^a^	1.16 (0.81–1.67)	2.02 (1.21–3.39)	10.37 (1.63–65.92)

Outcome variable: Age of diagnosis of hypertension.

*OR* odds ratio, *CI* confidence interval.

^a^No (reference category).

An adjusted model has been presented in Supplementary Table S1. The relationship between age at hypertension diagnosis and risk factor knowledge varies significantly when comparing the unadjusted and adjusted models. The impact of knowledge, especially about salt intake, on a subsequent diagnosis is still significant but becomes slightly weaker after adjustment.

## Discussion

In this cross-sectional study among a hypertensive population in rural Bangladesh, we found that the average age of hypertension of the participants was around 45.8 years, which aligns with some previous studies [18–21]. Moreover, uneducated people were diagnosed with hypertension at a later age. The results showed that those who do not know ‘extra salt intake’, ‘obesity’, ‘sedentary lifestyle’, ‘regular walking’, ‘watching health TV programs’, and ‘reading health newspapers’ diagnosed hypertension at an advanced age. In the case of age group analysis, those diagnosed with hypertension at the age of >65 have significantly less knowledge about healthy lifestyles.

Our study showed that hypertensive patients with no education, low income, and absence of a family history of hypertension were diagnosed with hypertension at a later age. All these factors indicated their inadequate knowledge and awareness about hypertension, which restrained them from the examination of BP and other diagnoses related to hypertension. As a result, although they have been diagnosed with hypertension, this late diagnosis pushes them to uncontrolled hypertension and other cardiovascular-related complications. In line with our study findings, previous studies among rural Bangladeshi populations also reported that the prevalence of undiagnosed hypertension increases with age (22.7% among those aged ≥60 years) [22]. A study by Gautam (2023) validates our findings, confirming that a positive history of hypertension, extra salt intake, and obesity, are significantly associated with a higher likelihood of developing hypertension at the age of 50 years of age in the rural population of Bangladesh [23]. Moreover, previous studies in poor resource settings mentioned that the prevalence of undiagnosed hypertension was higher among poor education attainment and lower socioeconomic status (SES) populations [24].

Our analysis regarding knowledge and hypertension diagnosis age demonstrated that knowledge about healthy lifestyle factors is associated with the age of hypertension diagnosis. Particularly, individuals who are aware of the health risks associated with consuming excess dietary salt are more likely to be diagnosed with hypertension at a later stage in life. This trend is particularly noticeable among people who are ≥65 years old. This later diagnosis may be due to lower awareness towards hypertension diagnosis and difficulties in accessing routine healthcare services in rural areas. Because of its asymptomatic nature, many people do not check their blood pressure regularly. Moreover, sometimes misconceptions, such as believing hypertension only affects older or overweight individuals, further contribute to delayed diagnosis. In rural areas, healthcare barriers, including limited access and reluctance to seek care, also prevent timely diagnosis. However, a previous study among the hypertensive Bangladeshi population mentioned that no significant variations in salt intake based on awareness [25]. This indicates that despite stakeholders’ knowledge distribution initiatives program, due to habitual preference, the people from this region continue taking extra salt intake. Individuals who do not watch health-related TV programs and read health newspapers tend to be diagnosed with hypertension at an older age. This may stem from a lack of awareness, preventive knowledge, and health monitoring practices.

Similarly, awareness of the health risks of obesity among ≥65 years old, sedentary lifestyles among 45–64 years old, and the importance of physical activity like walking and running among ≥65 years old shows a positive association with later diagnosis in older individuals [26–28]. This awareness likely encourages preventive behaviors, such as better dietary habits, increased physical activity, and regular health monitoring. Lack of practice of these health consciousnesses can result in an earlier onset of hypertension and later diagnosis. Furthermore, the study also found that engagement with health-related media, such as watching health programs on TV and reading health newspapers is associated with significantly higher odds of a delayed diagnosis of hypertension. Because, lack of engagement with health-related media diminishes awareness of risk factors and symptoms, leading to inadequate health monitoring and reduced knowledge. Without regular blood pressure checks, hypertension remains undiagnosed until symptoms or complications arise. In contrast, individuals with higher knowledge about healthy lifestyles tend to practice or follow it. So that, in the long term of their life there is a chance of spending a healthy life or later diagnosis of hypertension. In the case of smoking status, individuals who have quit smoking tend to be diagnosed with hypertension at an older age among those ≥65 years old compared to those <45 years old. It may be due to adopting healthier lifestyle choices, which may mitigate hypertension risk factors. Additionally, quitting smoking helps individuals recover from the damaging effects of smoking, leading to better health. Overall, a lack of knowledge about the determinants of hypertension leads to a later diagnosis.

### Strengths and limitations

To our knowledge, our study is the first to examine the association of age at hypertension diagnosis with knowledge about healthy lifestyles in a large population. Our study findings carry significant implications for public health interventions targeting hypertension awareness and prevention of possible hypertension-related complications. However, our study has several limitations. First, the cross-sectional nature of the study design limits to exploration of causal relationships. Further longitudinal investigations are required to explore the association between age at hypertension diagnosis and healthy lifestyle knowledge. Second, we identified the person had hypertension by measuring their BP multiple times and inquiring about their antihypertensive medication history rather than checking their actual diagnosis report from health professionals. Any additional diagnosis was not carried out to identify the secondary hypertension. Although it has a minimal impact on our findings as the previous study mentioned only 5–10% of hypertension patients may have secondary hypertension [29]. Third, recall bias may have influenced our findings, as participants might not accurately remember the exact age at which they were diagnosed with hypertension. This could affect the reliability of self-reported diagnosis age and its association with healthy lifestyle knowledge. Finally, our study subjects were limited to rural people. Comparison with urban people could identify different findings.

## Conclusion

The study reveals the key factors influencing the age of hypertension diagnosis among a rural Bangladeshi population. The study found that the average age of hypertension diagnosis of the participants is around 45.8 years. Moreover, the findings explored that individuals with lower educational levels, who lack awareness of healthy lifestyle factors such as extra salt intake, obesity, sedentary lifestyles, and limited engagement with health-related media, like watching health TV programs and reading health newspapers tend to be diagnosed with hypertension at a later age. Particularly, older adults who lack knowledge about lifestyle-related risks indicate a greater delay in diagnosis, which increases the risk of uncontrolled hypertension complications. This suggests that there is a significant relationship between knowledge of hypertension and hypertension diagnosis.

## Summary

### What is known about the topic

- Knowledge about healthy lifestyle for hypertension subjects helps to prevent further complications of hypertension.

- Inadequate knowledge in poor resource settings is considered an obstacle to timely hypertension diagnosis.

### What this study adds

- Individuals with poor knowledge about healthy lifestyles for hypertension are diagnosed with hypertension at later age.
- Our findings highlight the importance of integrated public health interventions to improve the knowledge about healthy lifestyles for hypertension among people in rural areas focusing on specific age groups helps in earlier hypertension diagnosis.

## Supplementary information

Supplementary file

## Data Availability

The data that support the findings of this study are available from the corresponding author upon reasonable request.
