# Supplementary material for: Healthy lifestyle knowledge and age at hypertension diagnosis: a primary health care based survey in Bangladesh
Source: J Hum Hypertens. 2025 Apr 25;39(6):448–56. doi: 10.1038/s41371-025-01019-3 (PMC12151846; doi:10.1038/s41371-025-01019-3)
Supplement: Supplementary file 1 — Supplementary file [file 41371_2025_1019_MOESM1_ESM.docx]

**Supplementary materials**

**Table S1.** Association between age of diagnosis of hypertension and knowledge about healthy lifestyle, Bangladesh

|  | **Age of diagnosis of hypertension (AOR, 95% CI)** | | |
| --- | --- | --- | --- |
| **Characteristics** | **45-54 years** | **55-64 years** | **≥65 years** |
| **Gender** |  |  |  |
| Female | 1.00 |  |  |
| Male | 2.85 (2.29-3.54) | 4.81 (3.75-6.18) | 6.94 (4.81-10.01) |
| **Education** |  |  |  |
| No education | 3.61 (2.56-5.11) | 6.14 (3.92-9.61) | 7.77 (3.66-16.48) |
| Primary | 2.12 (1.51-2.99) | 2.57 (1.64-4.04) | 1.76 (0.79-3.92) |
| Secondary | 1.21 (0.86-1.72) | 1.57 (1.00-2.47) | 0.98 (0.42-2.32) |
| Higher | 1.00 |  |  |
| **Body Mass Index (kg/m^2^)** |  |  |  |
| Underweight | 1.01(0.70-1.47) | 1.84 (1.27-2.67) | 2.45 (1.52-3.96) |
| Overweight | 0.69 (0.57-0.82) | 0.58 (0.46-0.72) | 0.42 (0.28-0.62) |
| Obese | 0.54 (0.40-0.73) | 0.46 (0.31-0.68) | 0.62 (0.34-1.11) |
| Normal Weight | 1.00 |  |  |
| **Blood pressure (mmHg)** |  |  |  |
| Poor | 0.93 (0.70-1.23) | 1.12 (0.77-1.63) | 0.85 (0.49-1.48) |
| Intermediate | 1.14 (0.88-1.48) | 1.85 (1.32-2.61) | 1.32 (0.80-2.18) |
| Ideal | 1.00 |  |  |
| **Expenditure quintile** |  |  |  |
| Q1 (poorest) | 1.42 (1.01-2.00) | 1.39 (0.92-2.09) | 0.95 (0.52-1.73) |
| Q2 | 1.00 (0.71-1.39) | 0.80 (0.53-1.20) | 0.46 (0.25-0.85) |
| Q3 | 1.05 (0.74-1.48) | 0.87 (0.57-1.33) | 0.58 (0.31-1.08) |
| Q4 | 0.96 (0.67-1.36) | 0.88 (0.57-1.35) | 0.60 (0.31-1.14) |
| Q5 (richest) | 1.00 |  |  |
| **Physical Exercise** |  |  |  |
| No | 1.19 (1.01-1.40) | 1.49 (1.22-1.81) | 1.87 (1.36-2.55) |
| Yes | 1.00 |  |  |
| **Smoking status** |  |  |  |
| Non-smoker | 1.00 |  |  |
| Ex-smoker | 1.06 (0.50-2.26) | 1.93 (0.93-4.04) | 1.81 (0.72-4.57) |
| Current-smoker | 0.63 (0.47-0.86) | 0.39 (0.27-0.57) | 0.21 (0.11-0.38) |
| **Family history of hypertension** |  |  |  |
| Yes | 0.77 (0.64-0.92) | 0.61 (0.48-0.77) | 0.55 (0.37-0.81) |
| No | 1.00 |  |  |
| **Knowledge** |  |  |  |
| Extra salt intake * | 1.04 (0.75-1.44) | 1.35 (0.95-1.92) | 1.85 (1.14-3.00) |
| Obesity * | 0.84 (0.69-1.02) | 1.10 (0.87-1.38) | 1.21 (0.85-1.73) |
| Sedentary lifestyle * | 0.99 (0.80-1.21) | 1.24 (0.96-1.59) | 1.01 (0.68-1.51) |
| Alcohol consumption* | 1.05 (0.84-1.33) | 1.06 (0.81-1.40) | 0.75 (0.49-1.14) |
| Walking/running * | 0.99 (0.82-1.19) | 0.80 (0.64-1.00) | 1.26 (0.89-1.80) |
| Sleep deprivation * | 1.03 (0.84-1.27) | 0.95 (0.75-1.21) | 0.84 (0.58-1.23) |
| Drug adherence * | 0.73 (0.60-0.88) | 0.79 (0.63-0.98) | 0.74 (0.52-1.05) |
| Monitoring BP * | 1.17 (0.95-1.44) | 1.03 (0.80-1.32) | 1.20 (0.81-1.78) |
| Watching health TV program * | 1.37 (1.00-1.87) | 0.97 (0.67-1.41) | 1.50 (0.73-3.10) |
| Reading health Newspaper* | 0.65 (0.41-1.02) | 1.06 (0.57-1.98) | 4.59 (0.58-36.25) |

* = No (reference category), AOR; Adjusted Odds Ratio, CI; Confidence Interval
